# FL118 Enhances Therapeutic Efficacy in Colorectal Cancer by Inhibiting the Homologous Recombination Repair Pathway through Survivin–RAD51 Downregulation

**DOI:** 10.3390/cancers16193385

**Published:** 2024-10-03

**Authors:** Jungyoun Kim, Yeyeong Jeong, You Me Shin, Sung Eun Kim, Sang Joon Shin

**Affiliations:** 1 Department of Medicine, Yonsei University College of Medicine, Seoul 03722, Republic of Korea; jungyounsum@yuhs.ac (J.K.); yyjeong1015@yuhs.ac (Y.J.); youme1129@yuhs.ac (Y.M.S.); sungeun5461@yuhs.ac (S.E.K.); 2 Songdang Institute for Cancer Research, Yonsei University College of Medicine, Seoul 03722, Republic of Korea; 3 Division of Medical Oncology, Department of Internal Medicine, Yonsei Cancer Center, Yonsei University College of Medicine, Seoul 03722, Republic of Korea

**Keywords:** FL118, irinotecan resistance, survivin, RAD51, DNA damage, colorectal cancer

## Abstract

**Simple Summary:**

Irinotecan is a widely used chemotherapy drug for colorectal cancer, but overcoming resistance to irinotecan remains a significant challenge. FL118, a drug similar to irinotecan, has shown promise as a new treatment option. FL118 functions by reducing the levels of a protein called survivin, which, in turn, decreases the expression of RAD51, a key protein involved in DNA repair. FL118 has been demonstrated to reduce cancer cell viability and overcome irinotecan resistance in colorectal cancer cells, suggesting it could serve as an effective treatment following irinotecan.

**Abstract:**

**Background/Objectives**: Irinotecan, a camptothecin (CPT) derivative, is commonly used as a first-line therapy for colorectal cancer (CRC), but resistance remains a significant challenge. This study aims to explore the therapeutic potential of FL118, another CPT derivative, with a focus on overcoming resistance to irinotecan. **Methods:** The effects of FL118 on CRC cells were evaluated, and bioinformatics analysis was performed on RNA-seq data. Transfection was conducted to observe the knockdown effect of survivin, and the in vivo efficacy of FL118 was assessed using a xenograft model. **Results**: FL118 induces apoptosis, G2/M arrest, and DNA damage. A notable mechanism of action of FL118 is a reduction in survivin levels, which downregulates the expression of RAD51, a key marker of homologous recombination, and attenuates DNA repair processes. Given that SN38 is the active metabolite of irinotecan, FL118 reduces cell viability and RAD51 in SN38-resistant LOVO cells. **Conclusions**: Our findings provide effective insights into the antitumor activity of FL118 and its potential as a therapeutic agent for overcoming irinotecan resistance in CRC.

## 1. Introduction

With recent advancements in colorectal cancer (CRC) management, a combination of surgery, radiotherapy, and chemotherapy has become the standard treatment. FOLFOX and FOLFIRI regimens, incorporating fluorouracil, leucovorin, oxaliplatin, and irinotecan, are key to treating metastatic CRC (mCRC) [1]. Additionally, targeted therapies, such as cetuximab and bevacizumab, have improved the outcomes in several patients with mCRC [2]. Despite these advances, drug resistance remains a significant challenge and has reduced the effectiveness of the current treatments over time [3].

Irinotecan faces limitations owing to its decreased intracellular concentration in cancers with ABCG2 overexpression, where it acts as a substrate for the ABCG2/BCRP efflux pump [4,5], a mechanism contributing to drug resistance. FL118, another camptothecin (CPT) analog, avoids expulsion from cancer cells via the ABCG2/BCRP pump and maintains its intracellular efficacy [6]. Importantly, FL118 inhibits apoptosis regulators, such as survivin, Mcl-1, and XIAP [7], and acts as a topoisomerase I (TOPO1) inhibitor, causing DNA damage [8]. Initial studies have demonstrated the efficacy of FL118 against CRC cells at low concentrations, suggesting its potential as a novel treatment option [9].

Furthermore, selecting appropriate candidates to enhance the anticancer effects associated with DNA damage is crucial. For example, olaparib, a poly (ADP-ribose) polymerase (PARP) inhibitor, induces DNA double-strand breaks (DSBs) and has demonstrated therapeutic efficacy in breast and ovarian cancers with BRCA1/2 gene mutations, which compromise DNA repair capabilities [10]. Consequently, we sought to elucidate the anticancer strategy of FL118, which predicts effective anticancer outcomes by inhibiting the expression of survivin and RAD51. This strategy simultaneously induces DNA damage and prevents DNA repair in CRC, thereby enhancing the potential for improved therapeutic outcomes.

This study investigated the mechanism of action of FL118 in CRC cell lines to explore its application as a new therapeutic option, identify possible synergistic drug combinations, and identify the patient groups that may benefit the most from its use. This effort seeks to enhance the effectiveness of CRC treatments, potentially contributing to improved clinical practice and patient outcomes.

## 2. Materials and Methods

### 2.1. Compounds and Antibodies

FL118 was supplied by Canget Biotekpharma LLC (Buffalo, NY, USA), and a portion of the material was provided to us. SN38 was purchased from MedChemExpress (Monmouth Junction, NJ, USA; HY-13704). Olaparib was purchased from Selleckchem Inc. (Houston, TX, USA; S1060). The primary antibodies used in this research were survivin (2808s), XIAP (2042s), Bcl-2 (sc-7382), GAPDH (sc-32233), β actin (sc-47778), cyclin B1 (sc-245), p-ATM (sc-47739), p-Histone H2AX (9718S), BRCA1 (sc-6954), Phospho-BRCA1 (Ser1524) (9009s), RAD51 (8875s, GTX70230), Ku70 (sc-17789), LIG4 (12695-I-AP), p53 (sc-126), p21 (sc-6246), p27 (sc-1641), cleaved caspase 3 (9664s), alpha Tubulin (sc-8035), and p-Histone H3 (Ser10) (06-570), purchased from diverse corporations, including Genetex (Irvine, CA, USA), Merck (Darmstadt, Germany), Abcam, Inc. (Cambridge, UK), Cell Signaling (Danvers, MA, USA), Proteintech (Chicago, IL, USA), and Santa Cruz Biotechnology (Santa Cruz, CA, USA).

### 2.2. Cell Culture and SN38-Resistant LOVO Cell Establishment

Cells purchased from the American Type Culture Collection (ATCC, Manassas, VA, USA) and the Korean Cell Line Bank (KCLB, Seoul, Republic of Korea) were grown in RPMI1640 (Cytiva, North Logan, UT, USA) and DMEM (Cytiva) supplemented with 10% fetal bovine serum (FBS), 100 μg/mL of streptomycin, and 100 U/mL of penicillin (Gibco, Waltham, MA, USA) at 37 °C and with 5% CO_2_ under humidified conditions. The SN38-resistant LOVO cell line was established through treatment with 10 nM SN38 and gradual increases in the final SN38 concentrations. A stable SN38-resistant LOVO cell line was maintained in RPMI1640 supplemented with 10% FBS, 100 μg/mL of streptomycin, and 100 U/mL of penicillin at 37 °C and with 5% CO_2_ under humidified conditions.

### 2.3. Cell Viability Assay and Normal Cell Toxicity

For the viability assay, 1.0 × 10^4^ cells were seeded into 96-well plates. After doubling the incubation time, FL118 and SN38 were added to the plates. After 48 h, 10 μL of CCK-8 reagent (Dojindo, Kumamoto, Japan) was added to each well. The absorbance of each well at 450 nm was estimated using an ELISA reader. The IC50 was calculated using GraphPad Prism 8.0.1 software (San Diego, CA, USA). To assess the short-term normal cell toxicity, FL118 was treated for 72 h using the CCK-8 assay. To assess long-term toxicity, 2 × 10^5^ cells were seeded into 6-well plates and treated with FL118 every two days. On day 8, the cell morphology was observed. Images were taken using the Operetta CLS (Perkin Elmer Inc., Waltham, MA, USA).

### 2.4. Western Blot

The cells were harvested with cold PBS and lysed with RIPA buffer containing proteinase inhibitors and phosphatase inhibitors. The supernatant was transferred, and the protein concentrations were calculated using the Bradford assay. Next, a sample containing SDS loading dye, PBS buffer, and identical amounts of the proteins was prepared. Acrylamide gels (6–12%) were used, and the proteins were separated by SDS–PAGE. The separated proteins on the acrylamide gels were transferred onto PVDF membranes, which were blocked with 5% skim milk. The membranes were incubated overnight with the primary antibodies at 4 °C, washed three times with 0.1% TBS-T, and incubated with horseradish peroxidase-conjugated secondary antibodies at room temperature. After washing, the protein bands were detected using an ECL reagent (EBP1071, ELPIS Biotech, Daejeon, Republic of Korea).

### 2.5. Flow Cytometry Analysis

For the cell cycle assay, cells were cultured in a 60 pi dish and treated with FL118. The cells were harvested, washed with PBS, and fixed with 70% EtOH for 2 h. The fixed cells were mixed with PI/RNase A staining buffer (BD Bioscience, Franklin Lakes, NJ, USA) and measured with a BD FACS LSR II using the BD FACSDiva 9.3 software.

### 2.6. Comet Assay

Before the assay, slides pre-coated with 1% low-gelling gel were prepared. The cells were treated with FL118 and harvested using trypsin and cold PBS. The cell concentration was measured using a hemocytometer, and the cells were diluted to 2 × 10^4^ cells/mL in PBS. Then, 400 μL of the cells was rapidly mixed with 1.2 mL of 1% low-gelling agarose gel and submerged in neutral lysis buffer overnight at 37 °C in the dark. After lysis, the samples were rinsed three times with TBE buffer for 30 min at room temperature. Next, the slides were submerged into an electrophoresis chamber with fresh TBE buffer and electrophoresed for 20 min. The samples were then rinsed with distilled water and immersed in 70% ethanol for 30 min at room temperature for fixation. The slides were dried in the dark and stained with DAPI (Sigma, St. Louis, MO, USA). An Operetta CLS (Perkin Elmer Inc., USA) was used for image acquisition. Image analysis was conducted using Cometscore2.0 software.

### 2.7. Immunofluorescence and Foci Analysis

Cells were plated into 96-well plates for the imaging experiments and fixed in 4% paraformaldehyde for 15 min at room temperature. After washing them twice with PBS, the cells were permeabilized using 0.1% Triton-X in PBS. They were then blocked with 3% BSA at room temperature for 30 min, incubated with the primary antibodies at 4 °C overnight, washed using PBS, and finally incubated with the Alexa Fluor 488- and Texas Red-labeled secondary antibodies for 1 h at room temperature. Their nuclei were stained using 1 μg/mL DAPI (Sigma, USA) after they were washed. Images were acquired using an Operetta CLS (Perkin Elmer Inc., USA). For foci analysis, spot analysis was performed using Harmony 4.9 software. The “select population” building block was added, and cells with more than 5 foci spots were counted.

### 2.8. qRT-PCR

Total RNA was extracted before qRT-PCR using Ribospin II (Geneall, Seoul, Republic of Korea), and its concentration was measured using a NanoDrop 1000 spectrophotometer (Thermo Scientific, Waltham, MA, USA). Then, 1000 ng of RNA was used for cDNA synthesis. Quantitative PCR was conducted using SYBR Green Master Mix following the instructions of the StepOnePlus PCR system. The primers used in this experiment were as follows: β actin forward: TTGCCGACAGGATGCAGAAG; β actin reverse: AGGTGGACAGCGAGGCCAGG; ATR forward: AGTAGCTTCCTTTCGCTCCAAA; ATR reverse: ACTGACTCCGGCCACTCCAT; ATM forward: GGTATAGAAAAGCACCAGTCCAGTATTG; ATM reverse: CGTGAACACCGGACAAGAGTTT; RAD51 forward: TTTGGAGAATTCGAACTGG; RAD51 reverse: TACATGGCCTTTCCTTCAC; survivin forward: CACCGCATCTCTACATTC; survivin reverse: GGTTTCCTTTGCATGG. The data analysis was conducted using 2^−ΔΔCt^ values.

### 2.9. Drug Combination Analysis

The cells were seeded into 96-well plate, and the cell viability was estimated to validate the synergistic effects of FL118 and olaparib. Using these data, we utilized the Combenefit 2.021 software. The data were analyzed using the HSA model and are illustrated as a graphical matrix to indicate the distribution of synergy.

### 2.10. Expression and RNA-Seq Data Analysis

Public CCLE expression data were obtained from the DepMap portal (https://depmap.org/portal/, accessed on 27 July 2022) and subsequently analyzed using the Data Explorer tool; the gene correlation was visualized with a scatter plot using R Studio (4.1.2). Pathway enrichment was analyzed with GSEA software (v_4.2.3), and “hallmark” and “KEGG” were applied to the gene set database. Enriched pathway, volcano plot, and heatmap analyses were performed using R Studio (4.3.2). For the pathway analysis, we additionally used the “wikipathways-20210310-gmt-Homo_sapiens.gmt” file (https://www.wikipathways.org/, accessed on 27 July 2022).

### 2.11. Transfection

Target-specific siRNA was transfected into the cells using Lipofectamine RNAiMAX reagent (Invitrogen, Waltham, MA, USA). The cells (40 × 10^4^ cells/well) were seeded into 6-well plates. The cell medium was replaced with non-antibiotic-containing medium. The Lipofectamine RNAiMAX reagent and the siRNA oligomers were diluted in Opti-MEM and incubated for 10 min at room temperature. Then, the cells were treated with 50 nM of the siRNA mixture (final concentration). We utilized AccuTarget™ Negative Control siRNA (SN-1002) from Bioneer (Daejeon, Republic of Korea) as the negative control. Predesigned siRNAs were purchased from Bioneer (Daejeon, Republic of Korea) as follows: si survivin #1: GUUUCAACUGUGCUCUUGU and ACAAGAGCACAGUUGAAAC; si survivin #2: GUUUUGAUUCCCGGGCUUA and UAAGCCCGGGAAUCAAAAC.

### 2.12. Xenografts

The animal experiments were approved by the Yonsei University Health System Institutional Animal Care and Use Committee. Six-week-old female BALB/c nude mice were purchased from Central Lab. Animal, Inc. (Seoul, Republic of Korea). Then, 2 × 10^6^ LOVO cells and SN38-resistant LOVO cells were mixed with Matrigel at a 1:1 ratio and implanted into each mice. Drug injection was initiated when the tumor volume reached approximately 200 mm^3^. The mice were randomly divided into treatment and control groups, which were intraperitoneally injected with 0.5 or 0.75 mpk FL118 and 40 mpk irinotecan, respectively, once a week. Tumor volume and body weight were measured three times per week. Tumor volume was calculated as follows:

Tumor volume = length (mm) × width^2^ (mm^2^) × 1/2 (1)

### 2.13. Immunohistochemistry

The xenograft tumors were harvested, fixed in 4% formalin overnight, and embedded into paraffin for IHC. The paraffin blocks were sectioned into slides, deparaffinized using xylene, and rehydrated using a graded series of ethanol with decreasing concentrations. Antigen retrieval was performed by incubating the slides in antigen retrieval buffer (Sigma, C9999, pH = 6.0) using an autoclave. The slides were incubated with methanol containing 1% H_2_O_2_ for 20 min to block endogenous peroxidase activity. After washing, the slides were blocked with 5% BSA. The primary antibodies used were Ki-67 (M7240, 1:100) and cleaved caspase 3 (9664S, 1:200), and the slides were incubated at 4 °C overnight. The slides were then incubated with the HRP–polymer-conjugated anti-rabbit/mouse secondary antibodies (Vector, Newark, CA, USA; MP-7500) for 1 h. After washing, DAB staining (Vector, SK-4100) was conducted, followed by counterstaining with hematoxylin (Tissueprotech, Gainesville, FL, USA; H08-500R). Quantification of the IHC results was performed using Image J 1.8.0 software, and the results were calculated using the following formula:

% area of staining = (IHC stained area)/(Total area) ×100%.(2)

### 2.14. Statistical Analysis

Statistical data were analyzed using GraphPad Prism 8.0.1 software. Data are presented as the mean ± S.D and SEM. A statistical comparison analysis was performed using an unpaired *t*-test and one-way ANOVA followed by Dunnett’s and Tukey’s tests. Significance is presented as follows: ns = not significant, * *p* < 0.05, ** *p* < 0.01, and *** *p* < 0.001.

## 3. Results

### 3.1. FL118 Triggered Apoptosis and G2/M Arrest by Suppressing Apoptosis Protein and Cell Cycle Protein

**Figure 1:**
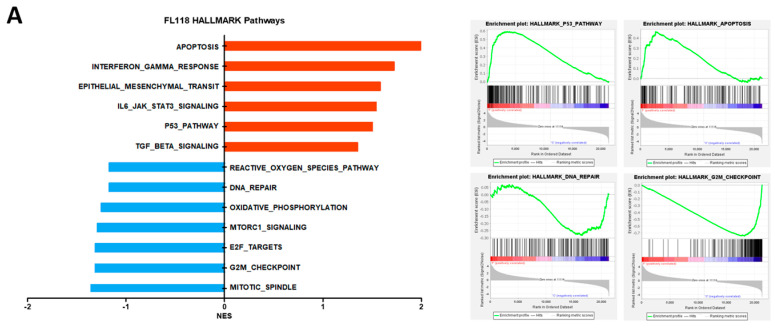

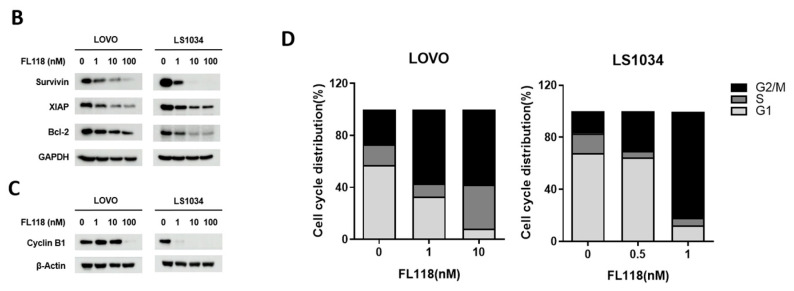
FL118 leads to apoptosis and G2/M cell cycle arrest. (**A**) Gene set enrichment analysis (GSEA) for FL118-treated LOVO parental cells. (**B**) The anti-apoptosis protein expression levels in LOVO and LS1034 cells treated with FL118 for 48 h were assessed using Western blot. (**C**) Cell cycle related protein levels when treating both with FL118 for 48 h, assessed by Western blot. (**D**) Cell cycle assay for flow cytometry in LOVO and LS1034 cells treated with FL118 for 48 h.

Prior research has demonstrated that FL118 diminishes the presence of apoptosis protein inhibitors (IAPs), specifically survivin, XIAP, and clAP2 [9], leading to apoptotic cell death. We conducted RNA sequencing (RNA-seq) on FL118-treated LOVO cells and subsequently performed gene set enrichment analysis (GSEA) to investigate the effects of FL118 on cancer cell death. FL118 enriched apoptosis and G2/M checkpoint pathways (Figure 1A). To confirm these findings, we evaluated the effect of FL118 on apoptosis and G2/M phase arrest in the LOVO and LS1034 cell lines. The administration of FL118 led to a reduction in IAPs (Figure 1B), which is associated with the induction of apoptotic cell death. FL118 also reduced cyclin B1 protein expression (Figure 1C), and the cell cycle analysis confirmed that FL118 induced G2/M phase arrest (Figure 1D). Compared to the control group, FL118 treated cells exhibited a reduction in pHH3 expression, with cells observed in a nondividing state (Figure S1). These results support previous findings that FL118 induces cell death in colon cancer cells by suppressing key genes involved in apoptosis and the G2/M checkpoint pathways.

### 3.2. FL118 Forms DSBs and Elevates Levels of Phosphorylated ATM and Phosphorylated γH2AX 

**Figure 2:**
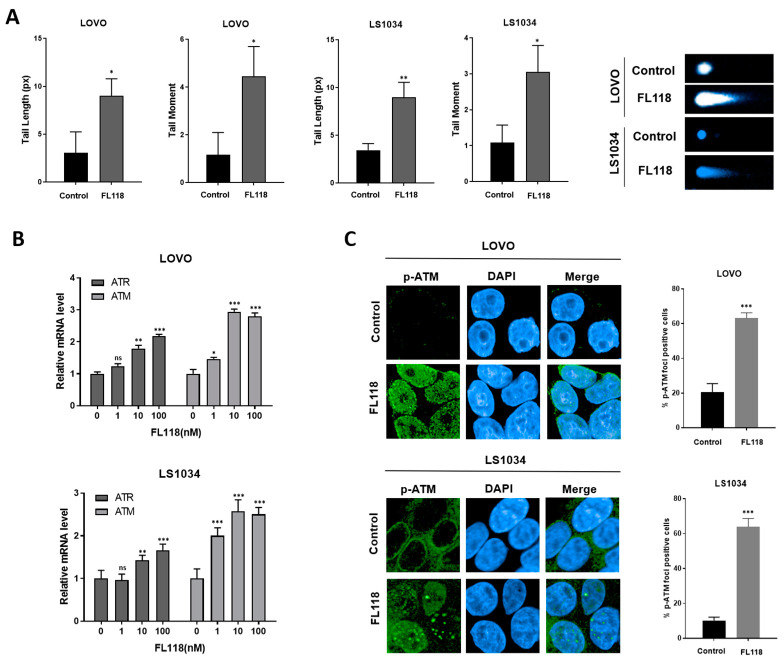

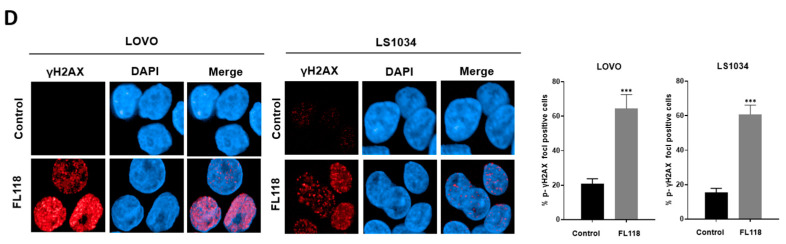
FL118 induced double-strand breaks leading to increased p-ATM and p-γH2AX. (**A**) Comet assay after 72 h of FL118 treatment shows increased DNA damage. Data are presented as the mean ± SD. (**B**) qRT-PCR analysis shows increased mRNA levels of ATR and ATM after treatment with FL118. (**C**) p-ATM foci assay indicates increased p-ATM levels after 48 h of FL118 treatment (10 nM for LOVO and 1 nM for LS1034). (**D**) p-γH2AX foci assay demonstrates increased p-γH2AX levels after 48 h of FL118 treatment (10 nM for LOVO and 1 nM for LS1034). Statistical significance was determined using an unpaired *t*-test for (**A**,**C**,**D**) and ANOVA–Dunnett’s test for (**B**). Significance was as follows: * *p* < 0.05, ** *p* < 0.01, and *** *p* < 0.001.

CPT compounds, which are known TOPO1 inhibitors, induce DNA damage. The GSEA demonstrated that FL118 affected the TP53 pathway and DNA repair (Figure 1A). We investigated whether FL118 caused DNA damage directly by assessing DNA fragmentation using a neutral comet assay. The length and moment of the tail nearly doubled (Figure 2A), and ATM and ATR mRNA levels were elevated upon treatment with FL118 (Figure 2B). These results indicated a more than three-fold increase in phosphorylated ATM (p-ATM) (Figure 2C). FL118 upregulated phosphorylated γH2AX (p-γH2AX), a DSB and DNA damage marker (Figure 2D). These results indicate that FL118 causes DSBs, increasing the expression of DNA damage markers.

### 3.3. FL118 Downregulates the Homologous Recombination (HR) Repair Gene RAD51

**Figure 3:**
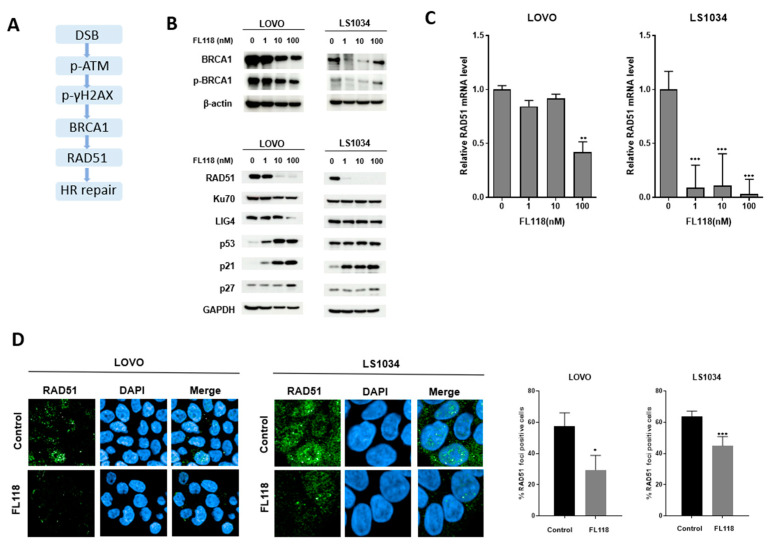
FL118 downregulated the homologous recombination (HR) repair gene RAD51. (**A**) DNA damage and repair pathway. (**B**) Western blot analysis for validation of the protein expression in LOVO and LS1034 cells treated with FL118 for 48 h. (**C**) qRT-PCR for RAD51 expression in LOVO and LS1034 cells treated with 1–100 nM FL118 for 48 h. Statistical value was determined by ANOVA–Tukey’s test. (**D**) RAD51 foci assay after treatment with FL118 for 48 h in LOVO and LS1034 cells. Statistical value was determined by an unpaired *t*-test. Significance was as follows: * *p* < 0.05, ** *p* < 0.01, and *** *p* < 0.001.

We further examined the impact of FL118 on homologous recombination (HR) repair following DSBs (Figure 3A). BRCA1, p-BRCA1, and RAD51 protein levels were low in the LOVO and LS1034 cells, with a reduction starting at 10 nM FL118 in LOVO and within the 1 nM-10 nM range in LS1034 (Figure 3B), and FL118 similarly downregulated the RAD51 mRNA levels (Figure 3C). Although the LIG4 expression decreased at 100 nM in the LOVO cells, the overall impact of FL118 on the NHEJ pathway was less pronounced compared to its more significant effect on RAD51, indicating that FL118 targets homologous recombination over NHEJ (Figure 3B). These findings show that FL118 selectively targets the HR repair system instead of NHEJ following DSBs. To confirm the effect of FL118 on HR repair, RAD51 foci formation was examined. The percentage of LOVO and LS1034 cells with RAD51 foci was considerably decreased (Figure 3D). These results revealed that FL118 not only causes DSBs (Figure 2) but also leads to a decrease in RAD51, thereby inhibiting HR repair.

### 3.4. FL118-Mediated Survivin Inhibition Downregulated RAD51

**Figure 4:**
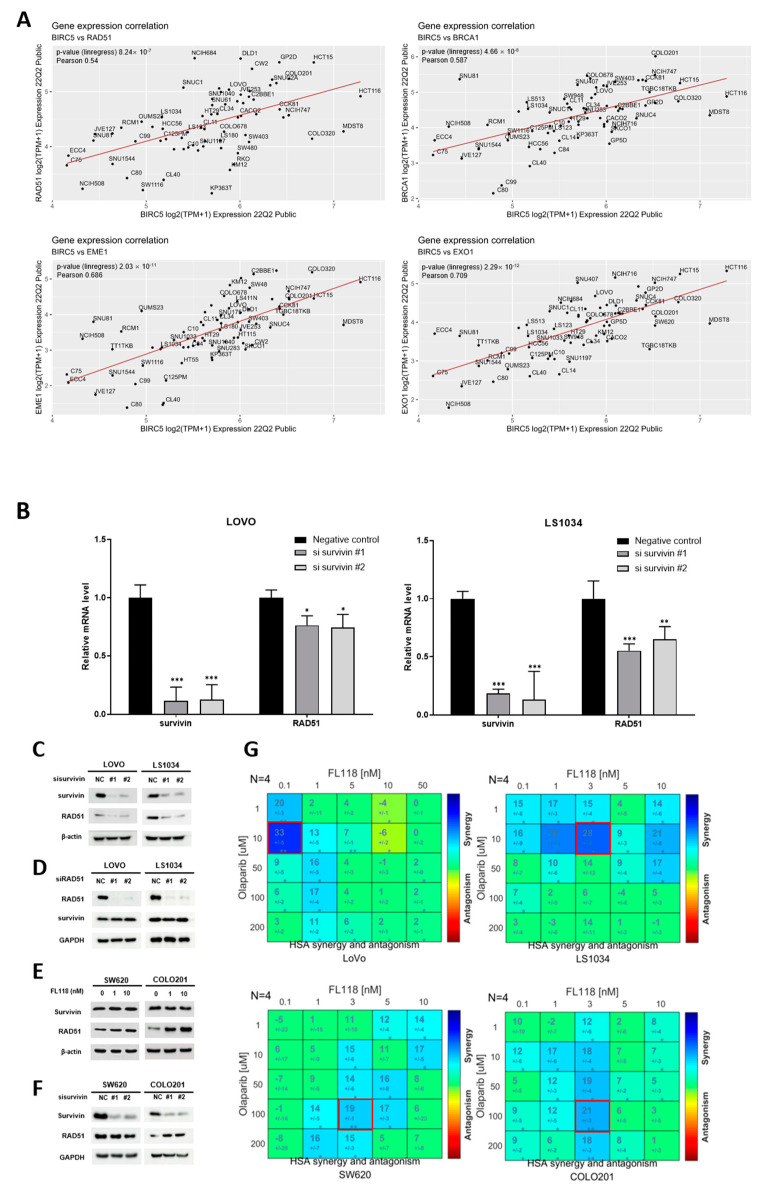
FL118-induced survivin inhibition affected RAD51. (**A**) Correlation of gene expression between BIRC5 and HR-repair-related genes (RAD51, BRCA1, EME1, and EXO1). Pearson’s correlation coefficient and the *p*-value are above each figure. (**B**) Survivin and RAD51 mRNA levels under transfection with si-survivin for 48 h. Statistical values were determined by ANOVA followed by Dunnett’s tests. Significance was as follows: * *p* < 0.05, ** *p* < 0.01, and *** *p* < 0.001. (**C**) Western blot analysis of LOVO and LS1034 cells treated with si-survivin for 48 h. (**D**) Western blot analysis of LOVO and LS1034 cells treated with si-RAD51 for 48 h. (**E**) Western blot analysis of SW620 and COLO201 cells treated with FL118 and (**F**) transected with si-survivin for 48 h. (**G**) Combenefit HSA score for estimating the synergistic effect between FL118 and olaparib treatment for 48 h in the LOVO, LS1034, SW620, and COLO201 cell lines.

Analysis of data from patients with COAD from the TCGA suggests targeting survivin as a therapeutic approach for cancer treatment (Figure S2A,B). A recent study found that decreased survivin levels in breast cancer cells hampered the function of HR-related genes and diminished HR activity [11]. YM155, a survivin inhibitor, reduced HR repair in esophageal squamous cell carcinoma [12], demonstrating the function of survivin in DNA damage response and repair pathways. We used the DepMap dataset to assess the correlation between survivin expression and HR-related gene expression in CRC. These findings confirmed the existence of a favorable association between BIRC5 (survivin) and crucial HR-related genes, including RAD51, BRCA1, EME1, and EXO1, in colon cancer cell lines (Figure 4A). Analysis of COAD patient data from the TCGA database produced comparable findings (Figure S2C). Thus, we demonstrated an association between survivin inhibition and RAD51 expression. RAD51 gene expression decreased when survivin was downregulated by siRNA transfection (Figure 4B). siRNA-mediated loss of survivin decreased the RAD51 protein levels (Figure 4C). Since siRAD51 did not alter the survivin levels, we hypothesized that survivin regulates RAD51 at the upstream level (Figure 4D).

Next, we assessed the extent of homologous recombination deficiency (HRD) in the two groups, classified according to their response to FL118-mediated survivin inhibition. This evaluation was conducted by combining FL118 with olaparib, which exerted synergistic effects during HRD. Based on these results, the LOVO and LS1034 cell lines were categorized into a survivin–RAD51 reduction group (Figure 1C and Figure 3B), whereas SW620 and COLO201 were classified into a stable survivin–RAD51 group (Figure 4E). In the stable survivin–RAD51 group, survivin depletion did not lead to RAD51 depletion (Figure 4F). The survivin–RAD51 reduction group exhibited higher HSA scores at lower olaparib doses than the stable survivin–RAD51 group (Figure 4G). These results were also observed in the LS513, HCT15, and SW403 cells (Figure S2E), showing HRD activation in the survivin–RAD51 reduction group. The combination of FL118 and olaparib suppressed both survivin and RAD51 proteins in the LOVO and LS1034 cells (Figure S2D). Overall, these findings indicate that a combination of FL118 and olaparib may be effective for treating cancer and that survivin depletion can serve as a biomarker for identifying suitable candidates for combination therapy.

### 3.5. FL118 Overcomes SN38 Resistance by Effectively Blocking the DNA Repair Mechanism

**Figure 5:**
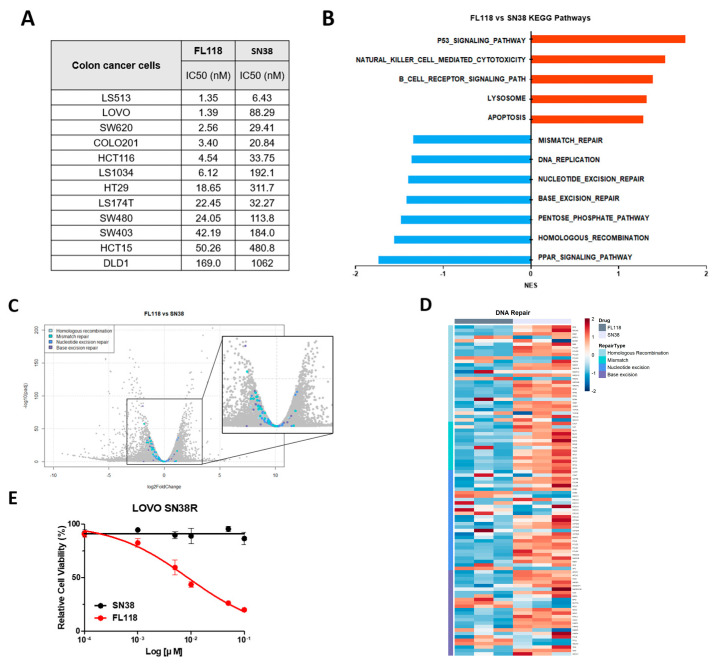

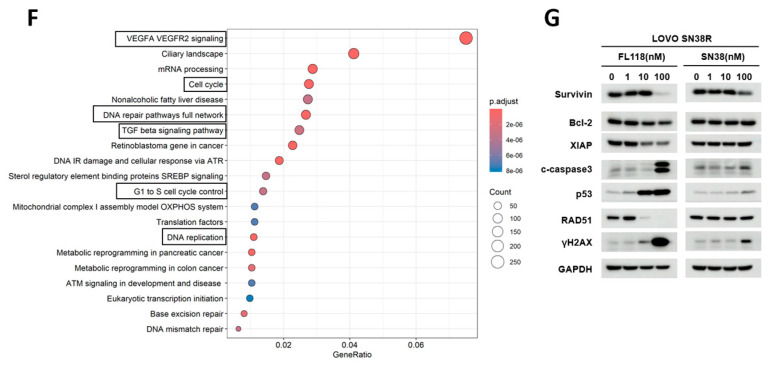
FL118 overcomes SN38 resistance by inhibiting the HR repair pathway. (**A**) IC50 values for FL118 and SN38, analyzed by CCK-8 assay. (**B**) GSEA pathway analysis, (**C**) volcano plot, and (**D**) heatmap for parental LOVO cells treated with FL118 and SN38. (**E**) CCK-8 assay of FL118 and SN38 in SN38-resistant LOVO cells. (**F**) Dot plot for enriched pathway analysis of SN38-resistant LOVO cells treated with FL118. (**G**) Western blot analysis of SN38-resistant LOVO cells treated with FL118 and SN38 for 48 h.

We aimed to evaluate the efficacy of FL118 in cell survival against that of SN38, the active form of irinotecan. The IC50 value of FL118 was lower than that of SN38 in various colon cancer cell lines (Figure 5A). The GSEA pathways showed that FL118 more pronouncedly downregulated DNA repair pathways, such as mismatch repair, nucleotide excision repair, base excision repair, and HR, than SN38 in the LOVO cells (Figure 5B). Genes associated with the repair pathway were positioned downstream of the volcano plot, and FL118 more strongly suppressed repair gene set expression than SN38 (Figure 5C,D). In summary, we hypothesized that FL118 would exhibit better antitumor efficacy than SN38, as it downregulated DNA-repair-related genes.

To assess the anticancer properties of FL118 in SN38-resistant cancer cells, we successfully developed a SN38-resistant LOVO cell line (LOVO SN38R) that exhibited SN38 resistance over a 6-month period. FL118 treatment significantly decreased the LOVO SN38R cell viability (Figure 5E). To understand the changes in the biological pathways during the development of SN38 resistance, we conducted an enriched pathway analysis. Our findings indicated that PI3K/AKT signaling genes, including PIK3R1, and focal-adhesion-pathway-related genes, such as FN1, ITGA1, and FYN, were upregulated in the LOVO SN38R compared to in the parental cells. Additionally, we verified the presence of potential resistance (Figure S3).

Next, we compared the effectiveness of the FL118 and SN38 treatments in the LOVO SN38R cells. Enriched pathway analysis showed that FL118 treatment decreased VEGFA signaling, cell cycle, and DNA repair pathways further than SN38 treatment (Figure 5F). Western blot analysis of cancer-related proteins, such as survivin, XIAP, cleaved caspase 3, and p53, provided insights into why FL118 was more potent than SN38 (Figure 5G). The FL118 treatment suppressed RAD51 activity and enhanced the levels of γH2AX in the LOVO SN38R cells (Figure 5G). These findings suggest that FL118 induces DNA damage while concurrently inhibiting repair mechanisms. These effects differed from those of SN38 and contributed to the anticancer properties of FL118.

### 3.6. FL118 Suppresses Tumor Growth in Both LOVO and LOVO SN38R Cell-Derived Xenograft Models

**Figure 6:**
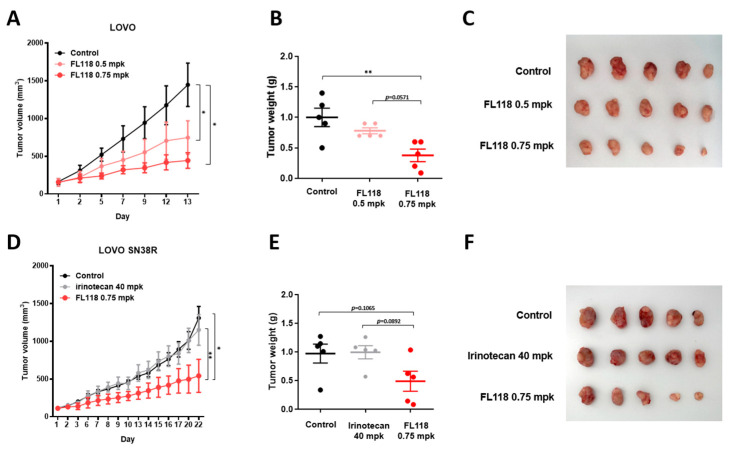
In vivo study of treating LOVO cell xenografts with FL118. (**A**) Tumor volume and (**B**) dot plot for tumor weight in LOVO cell-injected nude mice. (**C**) Image of LOVO cell xenograft tumors at the end of the experiment. (**D**) Tumor volume and (**E**) dot plot for tumor weight in SN38-resistant LOVO cell-injected nude mice. (**F**) Image of SN38-resistant LOVO cell xenograft tumors at the end of the experiment. Statistical values were determined by ANOVA–Tukey’s test. Data are presented as the mean ± SEM. Significance was as follows: * *p* < 0.05 and ** *p* < 0.01.

We conducted additional studies to determine its effectiveness in LOVO and LOVO SN38R cell-derived xenograft models. The LOVO xenograft models were administered with 0.5 and 0.75 mg/kg FL118 once weekly. FL118 inhibited tumor growth more than the control group (Figure 6A). The tumor weight reduced in the therapy group, with the average value decreasing more than twofold from 0.75 mpk (Figure 6B), and the tumor size was much smaller than that in the control group (Figure 6C). We also conducted experiments using LOVO SN38R cell xenografts to assess the ability of FL118 to overcome SN38 resistance. The LOVO SN38R-derived tumors were irinotecan-resistant, and the tumor size was reduced by nearly 40% after FL118 treatment (Figure 6D–F). The IHC analysis showed that the FL118 treatment decreased Ki-67 expression and increased c-caspase 3 in the LOVO SN38R xenografts, indicating reduced proliferation and enhanced apoptosis (Figure S4). In summary, FL118 may be a highly effective therapeutic agent for tumor growth inhibition in both parental and irinotecan-resistant cells in xenograft models.

## 4. Discussion

Our investigation made substantial progress in understanding the mechanism of action of FL118’s action against CRC. An important aspect of our research is the clear proof that FL118 causes DSBs, as shown by the increased p-ATM and p-γH2AX levels. This observation aligns with the established mechanisms of the DNA damage response [13,14]; however, FL118 demonstrated excellent efficacy and efficiency in this regard.

We identified key attributes of FL118’s function, including RAD51 suppression and resulting HR repair inhibition. This approach not only enhances the DNA damage caused by TOPO1 inhibitors, such as irinotecan, but also suggests that FL118 improves the therapeutic results by increasing DNA damage while simultaneously inhibiting the repair pathways activated in response to this damage, thereby enhancing apoptotic cell death. Thus, FL118 is an efficient option for overcoming the limitations of irinotecan, especially in the context of drug resistance.

Our research confirms previous findings that FL118 inhibits survivin and further demonstrates a direct correlation between survivin suppression and reduced RAD51 expression. This correlation highlights the possibility of using FL118 in combination with PARP inhibitors, providing a strategic benefit in CRC treatment. Previous studies have shown that irinotecan causes DNA damage and exhibits synergistic effects with PARP inhibitors [15,16,17,18] and have explored the combined effects of SN38 and olaparib [19]. Since FL118 reduced the survivin–RAD51 levels at lower concentrations than SN38, it should be more effective in combination therapy, with broader applications in cancer treatment. Olaparib has demonstrated efficacy in breast, ovarian, and pancreatic malignancies [20,21,22]; however, its usefulness in CRC has been restricted. Importantly, our results demonstrated that the synergy with olaparib was more effective in the group with reduced survivin levels after FL118 treatment. This finding suggests a promising strategy for future therapeutic applications using the survivin expression levels as a biomarker to identify patients who may benefit the most from this combination therapy. However, FL118 independently reduces multiple apoptotic proteins, including Mcl-1, XIAP, cIAP2, and MdmX, in addition to survivin [23]. Given that survivin interacts with various cell signaling pathways and growth factors [24], further studies are required to elucidate the interactions among the proteins reduced by FL118 and the subsequent alterations in cancer-related pathways, particularly those related to HR repair. Understanding these complex interactions will be crucial to optimizing the therapeutic application of FL118.

RNA sequencing data obtained from the LOVO SN38R cells indicated the upregulation of PI3K/Akt/mTOR pathway-associated genes (Figure S3). The PI3K pathway plays a role in the development of resistance to EGFR monoclonal antibodies [25]. Targeting the INHBA ligand of the TGF-β signaling pathway can effectively diminish TGF-β/PI3K/Akt signaling activity [26]. Collectively, these results suggest that FL118 could suppress the VEGFA and TGF-β pathways in these resistant cells (Figure 5F), thereby inhibiting PI3K/Akt signaling activation, which is a crucial factor in SN38 resistance.

Irinotecan, a derivative developed to overcome the low solubility and adverse effects of CPT, is a prodrug that is converted into a more potent anticancer agent, SN38 [27]. Recent studies have focused on utilizing CPT derivatives for advanced cancer treatments, such as in antibody–drug conjugate (ADC) development, to overcome their limitations. SN38, notable for its powerful anticancer effects even at low concentrations, has been utilized as a payload in ADCs [28,29]. Exatecan mesylate (DX-8951f), another CPT derivative, exhibits improved water solubility and potent TOPO1 inhibitory activity, making it well suited to ADC development because of its strong antitumor effects [30].

As a novel CPT payload for ADC development, FL118 treatment reduced the viability of LOVO SN38R cells, demonstrating effects comparable to those of exatecan (Figure S5). A xenograft mouse model indicated that FL118 is a highly potent ADC candidate for overcoming irinotecan resistance. The increased effectiveness of FL118, combined with its ability to overcome resistance, is similar to the shift in treatment from docetaxel to cabazitaxel as a therapeutic strategy for prostate cancer [31]. During this transition, drugs in the same category, such as irinotecan and FL118, offer therapeutic alternatives for resistant strains. Therefore, we suggest that FL118 can be used as an effective treatment option in irinotecan-resistant mCRC.

## 5. Conclusions

Our study indicates that FL118 activates distinct pathways that cause DNA damage, hinder HR repair, and overcome drug resistance in CRC. The therapeutic potential of FL118 was further emphasized by its efficacy in combination with PARP inhibitors and its promising application as an ADC payload. Although these findings are intriguing, further research is needed to completely understand the therapeutic potential of FL118, improve combination therapies, and identify biomarkers for its clinical use. Future research should focus on closing these gaps, facilitating FL118’s incorporation into clinical practice, and providing a promising solution for patients with CRC, particularly those who require additional therapeutic options due to treatment resistance.

## Data Availability

The relevant dataset is available on request from the authors.

## Supplementary Materials

The following supporting information can be downloaded at https://www.mdpi.com/article/10.3390/cancers16193385/s1. Supplementary Figure S1: FL118 induces cell cycle arrest in colon cancer cells ; Supplementary Figure S2: Analysis of survivin in COAD patients from TCGA data and the FL118–olaparib combination in colon cancer cells; Supplement Figure S3: Mechanisms of resistance in LOVO SN38R cells ; Supplement Figure S4: Ki-67 and c-caspase3 IHC analysis of LOVO SN38R xenografts ; Supplement Figure S5: Cell viability in LOVO SN38R cells ; Supplement Figure S6: Evaluation of FL118 toxicity on normal colon cells (CCD-18co) ; Supplement Figure S7: Original Western blot data.

## References

[1] Marques R.P., Duarte G.S., Sterrantino C., Pais H.L., Quintela A., Martins A.P., Costa J. (2017). Triplet (FOLFOXIRI) versus doublet (FOLFOX or FOLFIRI) backbone chemotherapy as first-line treatment of metastatic colorectal cancer: A systematic review and meta-analysis. Crit. Rev. Oncol. Hematol..

[2] Marques R.P., Godinho A.R., Heudtlass P., Pais H.L., Quintela A., Martins A.P. (2020). Cetuximab versus bevacizumab in metastatic colorectal cancer: A comparative effectiveness study. J. Cancer Res. Clin. Oncol..

[3] Panczyk M. (2014). Pharmacogenetics research on chemotherapy resistance in colorectal cancer over the last 20 years. World J. Gastroenterol..

[4] Martino E., Della Volpe S., Terribile E., Benetti E., Sakaj M., Centamore A., Sala A., Collina S. (2017). The long story of camptothecin: From traditional medicine to drugs. Bioorg. Med. Chem. Lett..

[5] Nakatomi K., Yoshikawa M., Oka M., Ikegamib Y., Hayasakab S., Sanob K., Shiozawaa K., Kawabataa S., Sodaa H., Ishikawad T. (2001). Transport of 7-ethyl-10-hydroxycamptothecin (SN-38) by breast cancer resistance protein ABCG2 in human lung cancer cells. Biochem. Biophys. Res. Comm..

[6] Ling X., Liu X., Zhong K., Smith N., Prey J., Li F. (2015). FL118, a novel camptothecin analogue, overcomes irinotecan and topotecan resistance in human tumor xenograft models. Am. J. Transl. Res..

[7] Li F. (2014). Anticancer drug FL118 is more than a survivin inhibitor: Where is the Achilles’ heel of cancer?. Am. J. Cancer Res..

[8] Han J.-Y., Lim H.-S., Shin E.S., Yoo Y.-K., Park Y.H., Lee J.-E., Jang I.-J., Lee D.H., Lee J.S. (2006). Comprehensive analysis of UGT1A polymorphisms predictive for pharmacokinetics and treatment outcome in patients with non-small-cell lung cancer treated with irinotecan and cisplatin. J. Clin. Oncol..

[9] Ling X., Cao S., Cheng Q., Keefe J.T., Rustum Y.M., Li F. (2012). A novel small molecule FL118 that selectively inhibits survivin, Mcl-1, XIAP and cIAP2 in a p53-independent manner, shows superior antitumor activity. PLoS ONE.

[10] Rose M., Burgess J.T., O’byrne K., Richard D.J., Bolderson E. (2020). PARP inhibitors: Clinical relevance, mechanisms of action and tumor resistance. Front. Cell Dev. Biol..

[11] Véquaud E., Desplanques G., Jézéquel P., Juin P., Barillé-Nion S. (2016). Survivin contributes to DNA repair by homologous recombination in breast cancer cells. Breast Cancer Res. Treat..

[12] Qin Q., Cheng H., Lu J., Zhan L., Zheng J., Cai J., Yang X., Xu L., Zhu H., Zhang C. (2014). Small-molecule survivin inhibitor YM155 enhances radiosensitization in esophageal squamous cell carcinoma by the abrogation of G2 checkpoint and suppression of homologous recombination repair. J. Hematol. Oncol..

[13] Lavin M.F., Kozlov S. (2007). ATM activation and DNA damage response. Cell Cycle.

[14] Podhorecka M., Skladanowski A., Bozko P. (2010). H2AX phosphorylation: Its role in DNA damage response and cancer therapy. J. Nucleic Acids.

[15] Yarchoan M., Myzak M.C., Johnson B.A., De Jesus-Acosta A., Le D.T., Jaffee E.M., Azad N.S., Donehower R.C., Zheng L., Oberstein P.E. (2017). Olaparib in combination with irinotecan, cisplatin, and mitomycin C in patients with advanced pancreatic cancer. Oncotarget.

[16] Augustine T., Maitra R., Zhang J., Nayak J., Goel S. (2019). Sensitization of colorectal cancer to irinotecan therapy by PARP inhibitor rucaparib. Investig. New Drugs.

[17] Williams S.M.G., Kuznicki A.M., Andrade P., Dolinski B.M., Elbi C., O’hagan R.C., Toniatti C. (2015). Treatment with the PARP inhibitor, niraparib, sensitizes colorectal cancer cell lines to irinotecan regardless of MSI/MSS status. Cancer Cell Int..

[18] Davidson D., Wang Y., Aloyz R., Panasci L. (2013). The PARP inhibitor *ABT*-888 synergizes irinotecan treatment of colon cancer cell lines. Investig. New Drugs.

[19] Tahara M., Inoue T., Sato F., Miyakura Y., Horie H., Yasuda Y., Fujii H., Kotake K., Sugano K. (2014). The use of Olaparib (AZD2281) potentiates SN-38 cytotoxicity in colon cancer cells by indirect inhibition of Rad51-mediated repair of DNA double-strand breaks. Mol. Cancer Ther..

[20] Griguolo G., Dieci M.V., Guarneri V., Conte P. (2018). Olaparib for the treatment of breast cancer. Expert Rev. Anticancer. Ther..

[21] Bixel K., Hays J.L. (2015). Olaparib in the management of ovarian cancer. Pharmacogenomics Pers. Med..

[22] Golan T., Hammel P., Reni M., Van Cutsem E., Macarulla T., Hall M.J., Park J.-O., Hochhauser D., Arnold D., Oh D.-Y. (2019). Maintenance olaparib for germline BRCA-mutated metastatic pancreatic cancer. N. Engl. J. Med..

[23] Li F. (2013). Discovery of survivin inhibitors and beyond: FL118 as a proof of concept. Int. Rev. Cell Mol. Biol..

[24] Kanwar J.R., Kamalapuram S.K., Kanwar R.K. (2011). Targeting survivin in cancer: The cell-signalling perspective. Drug Discov. Today.

[25] Sforza V., Martinelli E., Ciardiello F., Gambardella V., Napolitano S., Martini G., della Corte C., Cardone C., Ferrara M.L., Reginelli A. (2016). Mechanisms of resistance to anti-epidermal growth factor receptor inhibitors in metastatic colorectal cancer. World J. Gastroenterol..

[26] Xiao Q., Xiao J., Liu J., Liu J., Shu G., Yin G. (2022). Metformin suppresses the growth of colorectal cancer by targeting INHBA to inhibit TGF-β/PI3K/AKT signaling transduction. Cell Death Dis..

[27] Garcia-Carbonero R., Supko J.G. (2002). Current perspectives on the clinical experience, pharmacology, and continued development of the camptothecins. Clin. Cancer Res..

[28] Yaghoubi S., Karimi M.H., Lotfinia M., Gharibi T., Mahi-Birjand M., Kavi E., Hosseini F., Sepehr K.S., Khatami M., Bagheri N. (2020). Potential drugs used in the antibody–drug conjugate (ADC) architecture for cancer therapy. J. Cell Physiol..

[29] Starodub A.N., Ocean A.J., Shah M.A., Guarino M.J., Picozzi V.J., Vahdat L.T., Thomas S.S., Govindan S.V., Maliakal P.P., Wegener W.A. (2015). First-in-human trial of a novel anti-Trop-2 antibody-SN-38 conjugate, sacituzumab govitecan, for the treatment of diverse metastatic solid tumors. Clin. Cancer Res..

[30] Nakada T., Masuda T., Naito H., Yoshida M., Ashida S., Morita K., Miyazaki H., Kasuya Y., Ogitani Y., Yamaguchi J. (2016). Novel antibody drug conjugates containing exatecan derivative-based cytotoxic payloads. Bioorg. Med. Chem. Lett..

[31] Buck S.A., Koolen S.L., Mathijssen R.H., de Wit R., van Soest R.J. (2021). Cross-resistance and drug sequence in prostate cancer. Drug Resist. Updat..

