# Supplementary material for: FL118 Enhances Therapeutic Efficacy in Colorectal Cancer by Inhibiting the Homologous Recombination Repair Pathway through Survivin–RAD51 Downregulation
Source: Cancers (Basel). 2024 Oct 3;16(19):3385. doi: 10.3390/cancers16193385 (PMC11475853; doi:10.3390/cancers16193385)

Supplement Figure S1

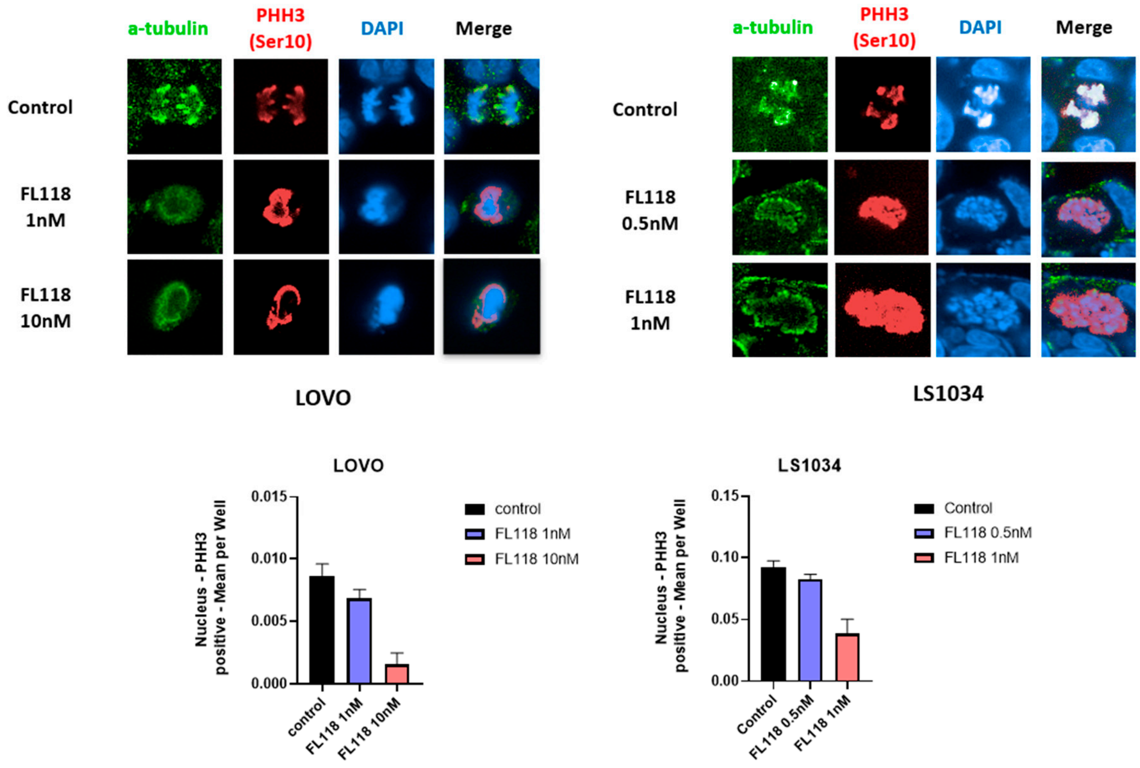

Supplement Figure S1. FL118 induces cell cycle arrest in colon cancer cells.

Double-staining of pHH3(Ser10) and alpha-tubulin to assess cell cycle dysregulation caused by FL118. FL118-treated cells showing a reduction in pHH3 expression.

Supplement Figure S2

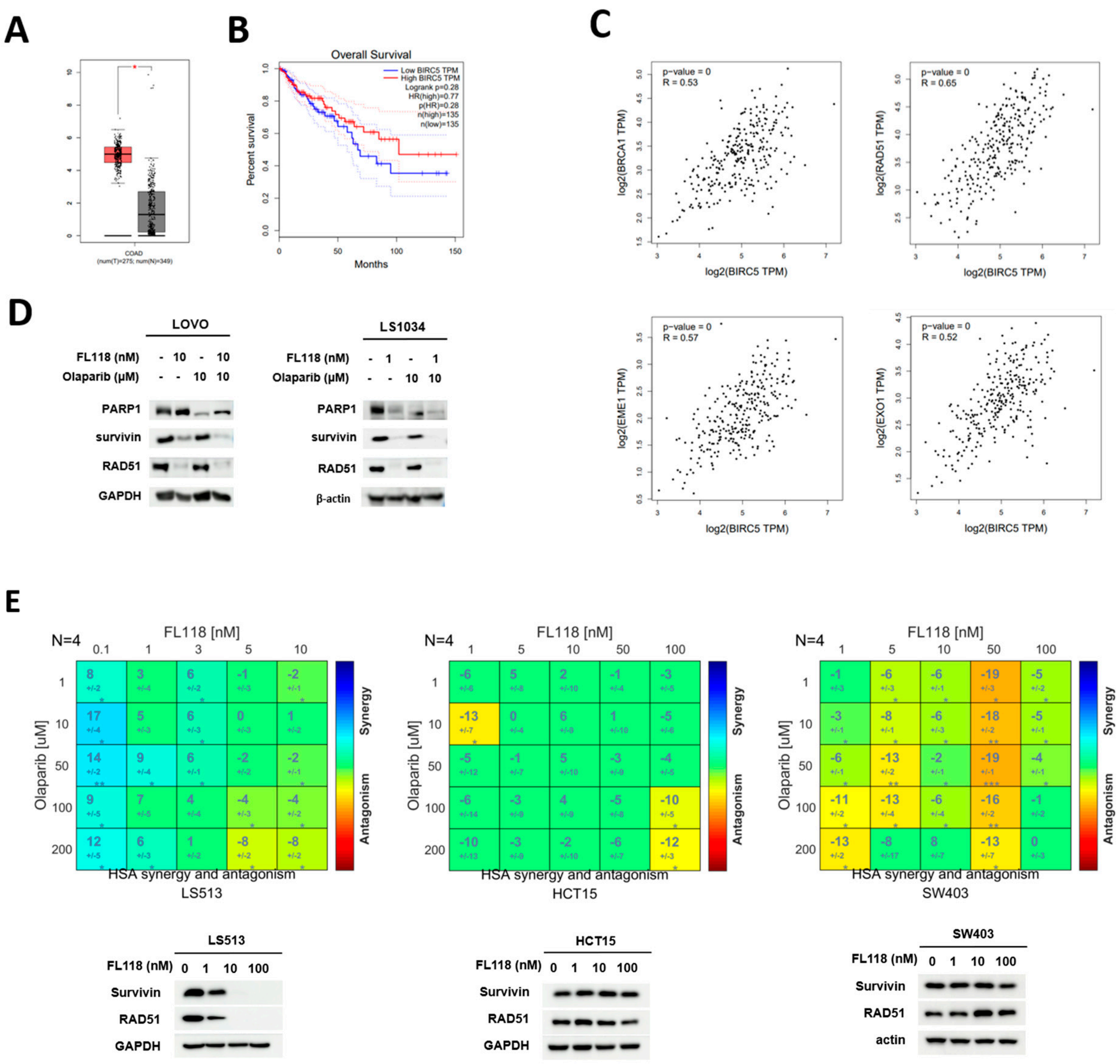

Supplement Figure S2. Analysis of Survivin in COAD Patients from TCGA Data and the FL118-Olaparib Combination in colon cancer cells.

(A) BIRC5(survivin) expression in TCGA-COAD patients and (B) survival curve in TCGA-COAD patients, obtained from GEPIA (C) Correlation of expression between BIRC5 and HR repair genes (RAD51, BRCA1, EME1, and EXO1) in TCGA-COAD patients, obtained from GEPIA. Pearson Correlation Coefficient and p-value are above each figures. (D) Combination effect of FL118 and olaparib subjected to Western blot analysis with indicated antibodies. (E) Combenefit score to estimate synergistic effect between FL118 and Olaparib for 48hours in LS513, HCT15 and SW403, following western blot with survivin and RAD51.

Supplement Figure S3

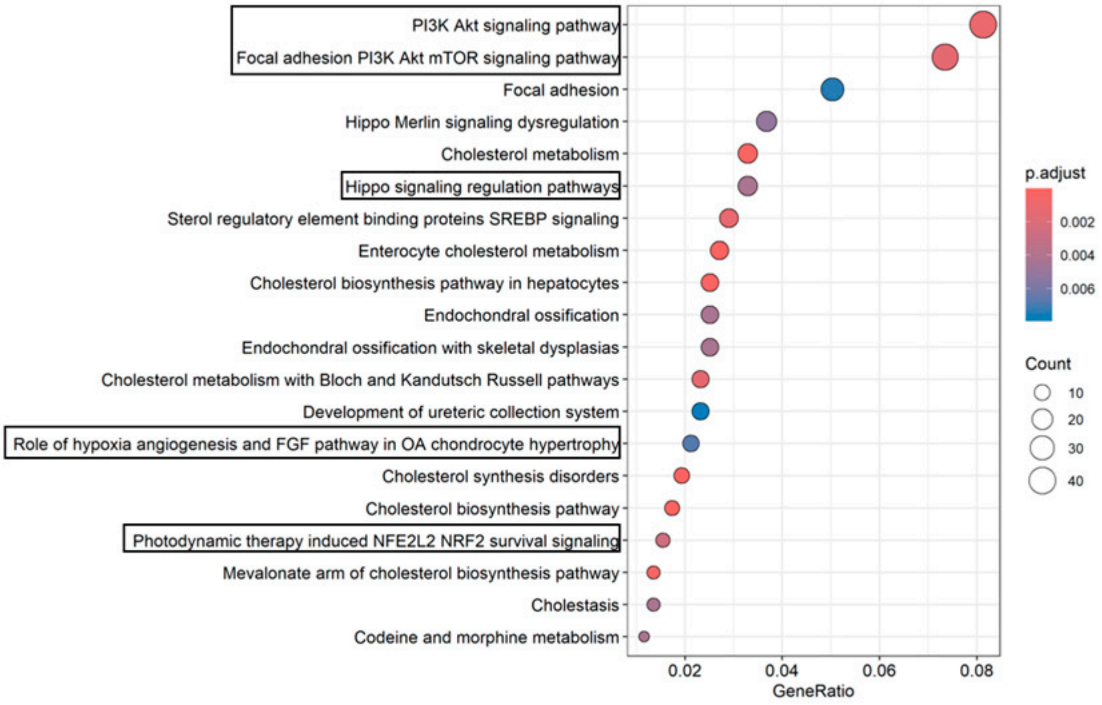

Supplement Figure S3. Mechanisms of resistance in LOVO SN38R cells.

Dotplot for enriched pathway analysis compared with LOVO parental and LOVO SN38 resistant RNA-seq data.

Supplement Figure S4

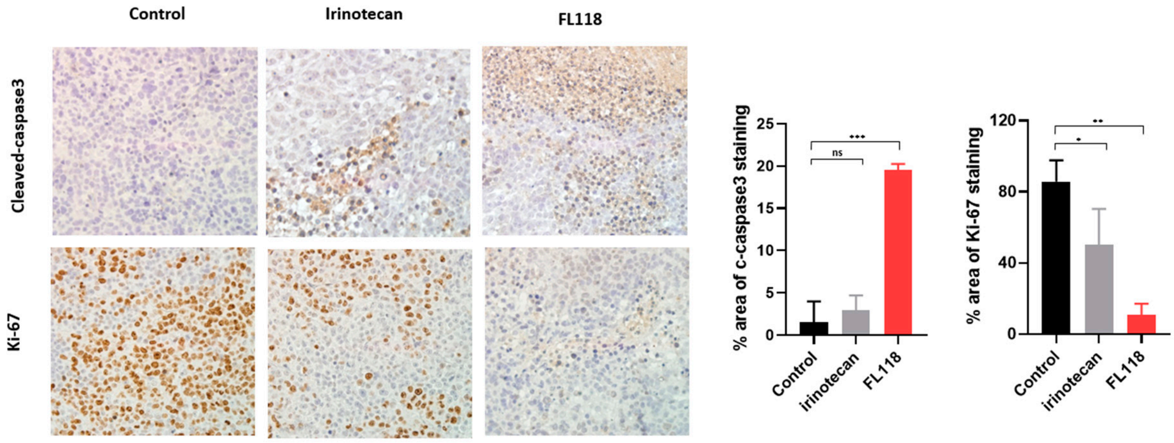

Supplement Figure S4. Ki-67 and c-caspase3 IHC analysis of LOVO SN38R xenografts. Immunohistochemistry (IHC) was performed on paraffin-embedded sections from LOVO SN38R xenografts to evaluate the effects of FL118 treatment on cell proliferation and apoptosis. Quantification of IHC results was calculated as (IHC stained area) / (Total area) * 100%. Statistical value was determined by ANOVA-Dunnett's.

Supplement Figure S5

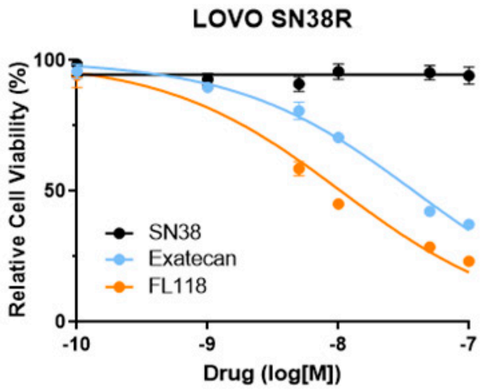

	IC50 [nM] in LOVO SN38R
SN38	NA
FL118	10.50
Exatecan	38.03

Supplement Figure S5. Cell viability in LOVO SN38R cells.

Cell viability assay with CPT analogues, treated for 48hours, in LOVO SN38 resistant cell.

Supplement Figure S6

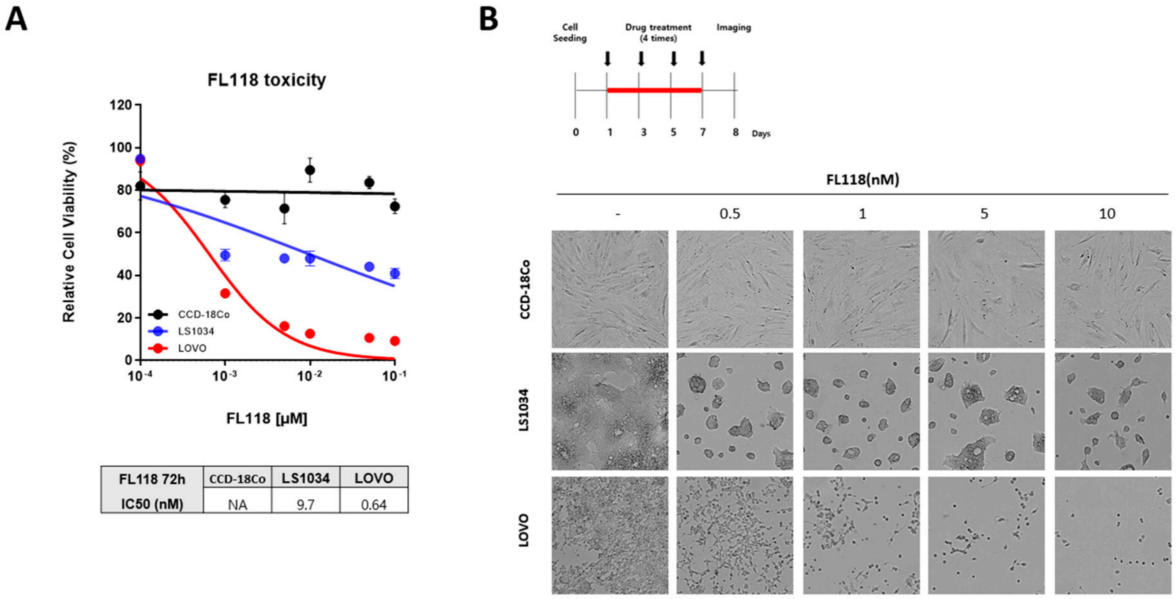

FL118 72h	CCD-18Co	LS1034	LOVO
IC50 (nM)	NA	9.7	0.64

Supplement Figure S6. Evaluation of FL118 toxicity on normal colon cells (CCD-18co) (A) Normal colon cells (CCD-18co) and colon cancer cells (LOVO and LS1034) were treated with FL118 at concentrations ranging from 0.1nM to 100nM for 72 hours. Cell viability was assessed using a CCK-8 assay. (B) Representative cell images over 8 days of FL118 treatment at different concentrations.

Supplement Figure S7: Original Western blot data

In Figure 1B and C

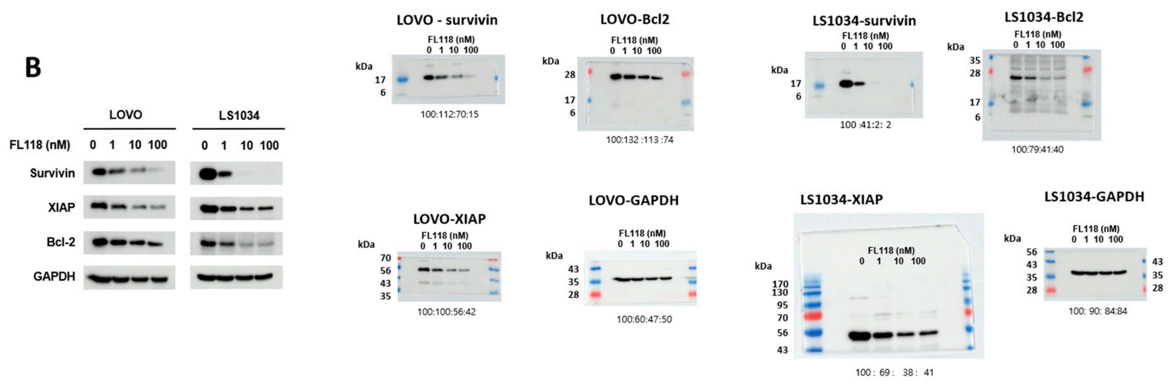

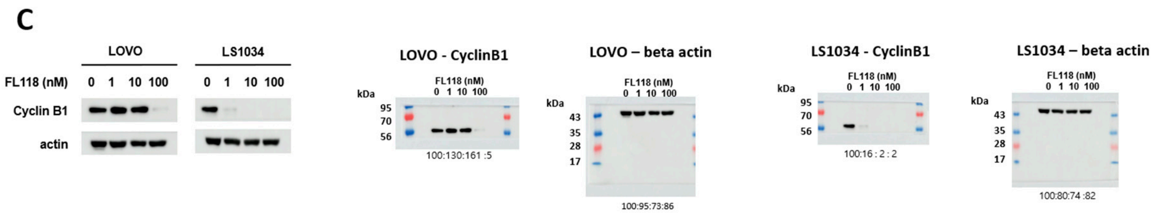

In Figure 3B

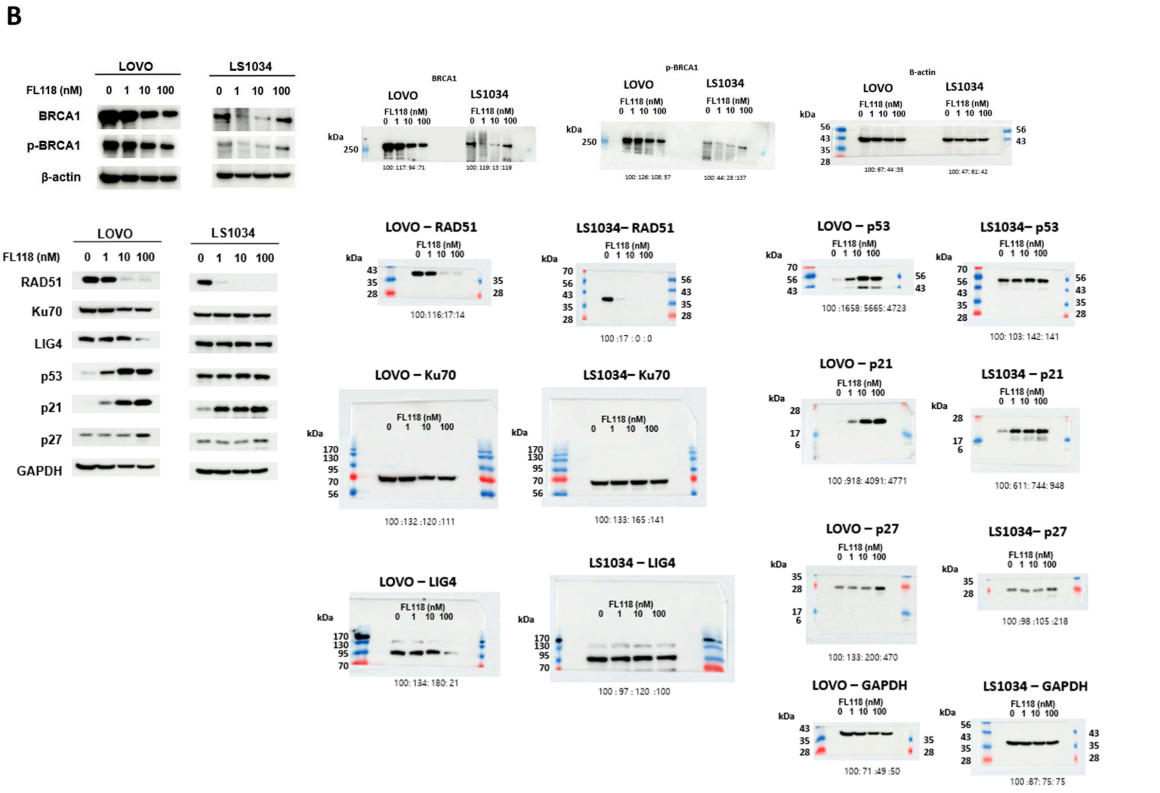

In Figure 4C,D,E and F

C

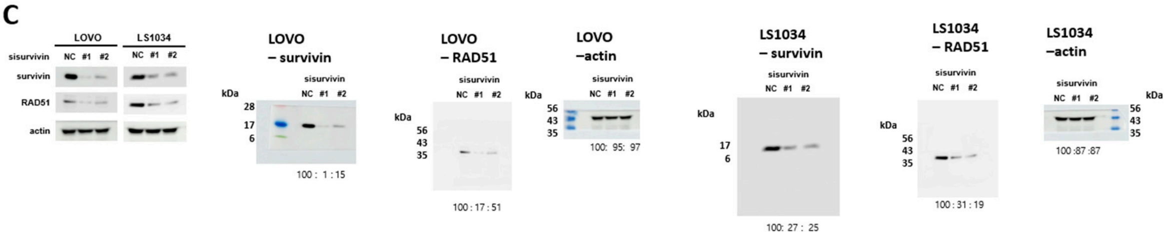

D

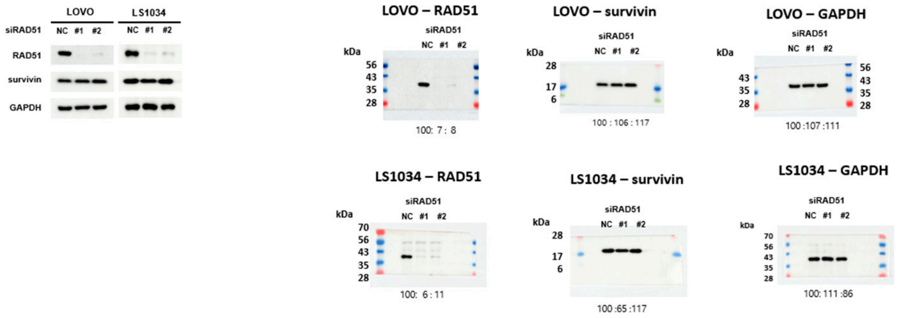

E

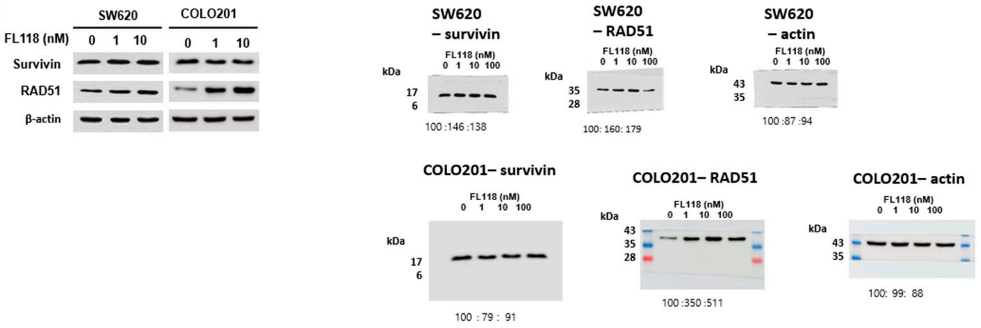

F

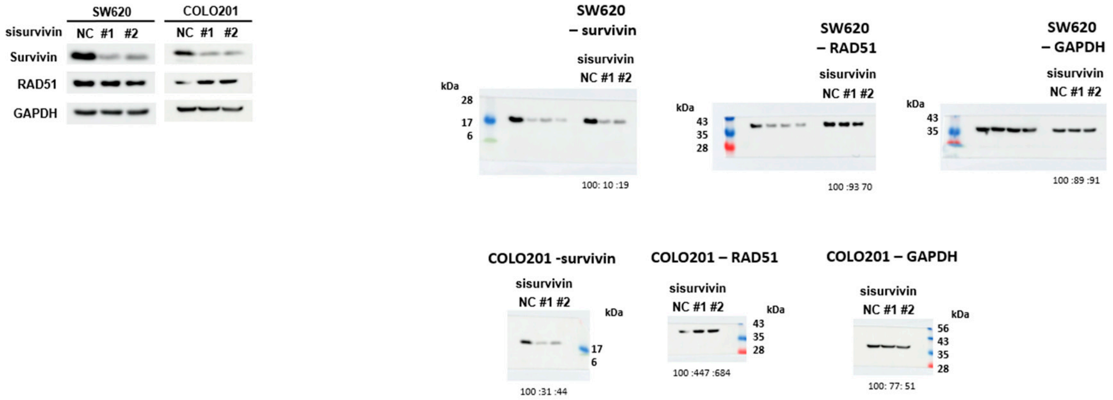

In Figure 5G

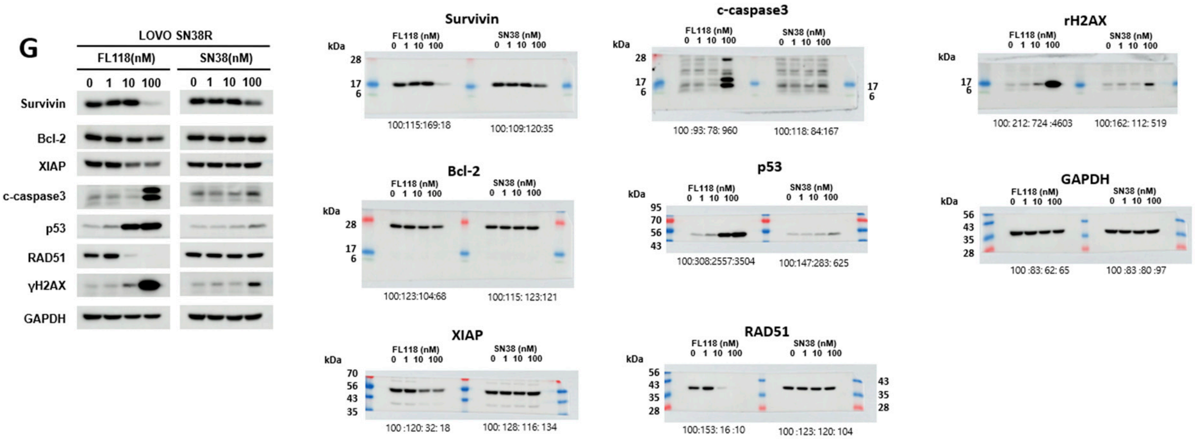

In Supplement Figure S2D and E

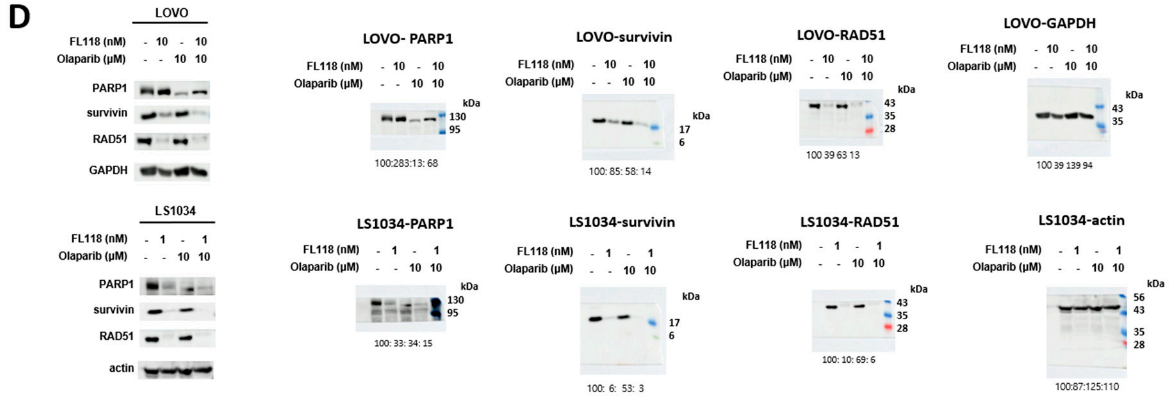

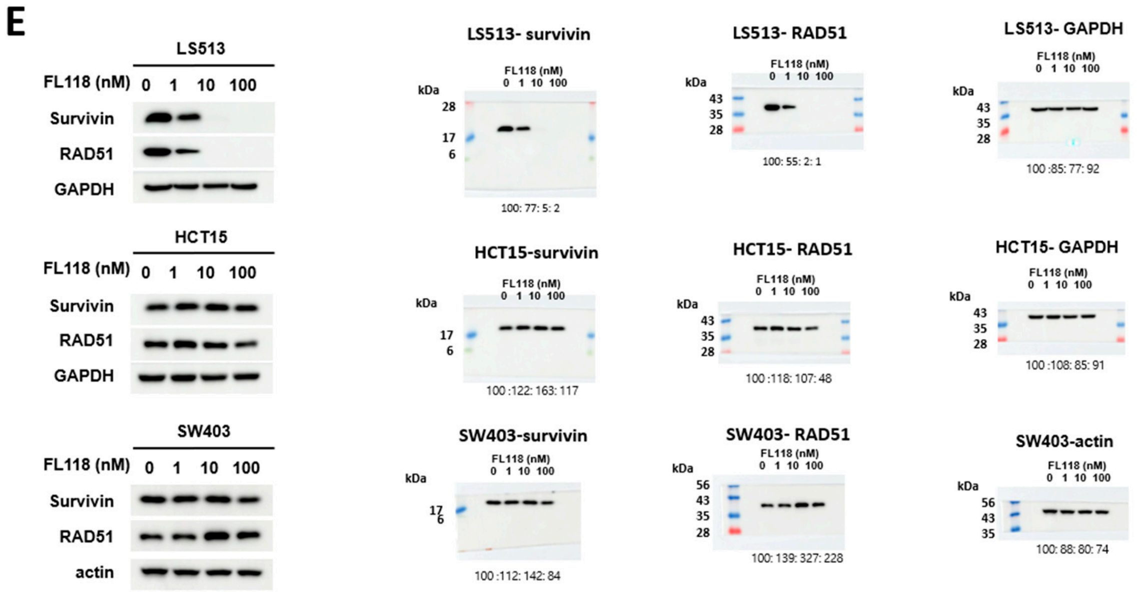

Supplement: Supplementary file 1 [file cancers-16-03385-s001.zip › cancers-3174639-supplementary.pdf]
